# Bone Biomarkers Help Grading Severity of Coronary Calcifications in Non Dialysis Chronic Kidney Disease Patients

**DOI:** 10.1371/journal.pone.0036175

**Published:** 2012-05-02

**Authors:** Marion Morena, Isabelle Jaussent, Aurore Halkovich, Anne-Marie Dupuy, Anne-Sophie Bargnoux, Leila Chenine, Hélène Leray-Moragues, Kada Klouche, Hélène Vernhet, Bernard Canaud, Jean-Paul Cristol

**Affiliations:** 1 Laboratoire de Biochimie, CHRU de Montpellier, Montpellier, France; 2 Institut de Recherche et de Formation en Dialyse, Montpellier, France; 3 UMR 204, Nutripass, Université Montpellier I, Montpellier, France; 4 INSERM, U1061, and Université Montpellier I, Montpellier, France; 5 Service de Néphrologie-Hémodialyse et Soins Intensifs, CHRU de Montpellier, Montpellier, France; 6 Service de Réanimation Métabolique, CHRU de Montpellier, Montpellier, France; 7 Service de Radiologie, CHRU de Montpellier, Montpellier, France; Harvard Medical School, United States of America

## Abstract

**Background:**

Osteoprotegerin (OPG) and fibroblast growth factor-23 (FGF23) are recognized as strong risk factors of vascular calcifications in non dialysis chronic kidney disease (ND-CKD) patients. The aim of this study was to investigate the relationships between FGF23, OPG, and coronary artery calcifications (CAC) in this population and to attempt identification of the most powerful biomarker of CAC: FGF23? OPG?

**Methodology/Principal Findings:**

195 ND-CKD patients (112 males/83 females, 70.8 [27.4–94.6] years) were enrolled in this cross-sectional study. All underwent chest multidetector computed tomography for CAC scoring. Vascular risk markers including FGF23 and OPG were measured. Logistic regression analyses were used to study the potential relationships between CAC and these markers.

The fully adjusted-univariate analysis clearly showed high OPG (≥10.71 pmol/L) as the only variable significantly associated with moderate CAC ([100–400[) (OR = 2.73 [1.03;7.26]; p = 0.04). Such association failed to persist for CAC scoring higher than 400. Indeed, severe CAC was only associated with high phosphate fractional excretion (FEPO_4_) (≥38.71%) (OR = 5.47 [1.76;17.0]; p = 0.003) and high FGF23 (≥173.30 RU/mL) (OR = 5.40 [1.91;15.3]; p = 0.002). In addition, the risk to present severe CAC when FGF23 level was high was not significantly different when OPG was normal or high. Conversely, the risk to present moderate CAC when OPG level was high was not significantly different when FGF23 was normal or high.

**Conclusions:**

Our results strongly suggest that OPG is associated to moderate CAC while FGF23 rather represents a biomarker of severe CAC in ND-CKD patients.

## Introduction

Coronary artery calcifications (CAC) are recognized as a strong predictor of all-cause and cardiovascular mortality in hemodialysis (HD) patients [1]. Recent studies now provide strong evidence for the risk of death with a high CAC score in chronic kidney disease (CKD) patients before onset of dialysis [2], [3]. Presence of CAC has been associated with numerous traditional risk factors including aging, hypertension and diabetes as well as with non traditional risk factors including mineral metabolism disorders, hyperparathyroidism, inflammation, osteoprotegerin (OPG) and more recently fibroblast growth factor 23 (FGF23) [4].

OPG is a bone regulating protein belonging to the TNF receptor superfamily. With its ligand, the receptor activator of nuclear factor kB ligand (RANKL), it regulates important aspects of osteoclast/osteoblast formation [5]. RANKL increases the pool of active osteoclasts by activating its specific receptor RANK located partly on osteoclastic cells, thus increasing bone resorption, whereas OPG, which neutralizes RANKL, has opposite effects. RANKL and OPG are produced by bone marrow derived stromal cells and osteoblasts and are regulated by various calcitropic cytokines, hormones, and drugs. The actions of OPG and RANKL in the vasculature and heart are still unclear but no doubt exists about the strong association of OPG with the prevalence of vascular calcifications in predialysis [6] and HD patients [7].

FGF23 is the main regulator of phosphate homeostasis. It is produced by osteocytes in response to hyperphosphatemia and exerts its effects on its receptor, the Klotho-FGFR1c heterodimer, in the kidney where it inhibits the expression of Na-Pi cotransporters resulting in hyperphosphaturia. Literature data are more controversial regarding FGF23 association with vascular calcifications according to the stage of CKD. Relationships between FGF23 and aortic calcifications [8] or peripheral vascular calcifications [9], [10] are well documented in HD patients. By contrast, studies in non dialysis CKD (ND-CKD) patients could not evidence any association after multivariable adjustment [11] but rather relationships to atherosclerosis [12] and left ventricular hypertrophy [11], [13].

We therefore investigated in this cross-sectional study the relationships between FGF23, OPG and CAC prevalence in a population of ND-CKD patients and attempted to identify the most powerful biomarker(s) of CAC: FGF23? OPG?

## Methods

### Ethics Statement

The study was conducted according to the principles of the Declaration of Helsinki and in compliance with International Conference on Harmonization/Good Clinical Practice regulations. According to the French Law, the study has been registered at “Ministère de la Santé et des Solidarités” after approval by the Montpellier University Hospital's ethics committee (Comité de Protection des Personnes Sud Méditerranée IV) with the following number 2006-A00416-45. All patients gave their written informed consent.

### Subjects

One hundred and ninety five ND-CKD patients at various stages of kidney disease, issued from the outpatient general nephrology consultation of the Montpellier Lapeyronie university hospital, were enrolled in this cross-sectional study. Inclusion criteria were age≥18 years and presence of CKD defined by glomerular filtration rate (GFR) in agreement with the National Kidney Foundation [14].

Causes of CKD were glomerulonephritis (n = 25), cystic renal disease (n = 14), diabetic nephropathy (n = 21), diabetic and hypertensive nephropathy (n = 21), angiosclerosis and hypertensive nephropathy (n = 79), infectious/obstructive interstitial nephropathy (n = 4), renal neoplasia (n = 1), genetic/congenital cause (n = 1), necrotizing angiitis (n = 3), unknown cause (n = 9), other cause (n = 17).

Detailed medical history including age, gender, weight, height, diabetes mellitus, hypertension, past or current smoking, presence of atherosclerotic cardiovascular disease was recorded.

Existence of hypertension was defined by brachial blood pressure higher or equal to 130/80 mmHg and/or by a current antihypertensive treatment.

Presence of atherosclerotic cardiovascular disease was defined by the presence of at least one of the three following manifestations: coronary heart disease (CHD), cerebrovascular disease (CVD) or peripheral vascular disease (PVD). *CHD* was defined as documented angina pectoris or history of myocardial infarction. Angina pectoris was described as chest pain arising at exertion and disappearing with nitroglycerine or rest. The diagnosis may also have been made by a positive evaluation in nuclear medicine or a coronary stenosis >75% of luminal diameter evidenced by angiography. Myocardial infarction was defined as clinical symptoms, such as chest pain or dyspnea, associated with abnormal electrocardiogram and elevated troponins. *CVD* was defined as previous clinical cerebral disease (transient ischemic attack or stroke) or the presence of atheromatous plaques on internal carotid arteries. *PVD* included clinical symptoms such as intermittent claudication, abolished peripheral pulses or diminished arterial pulses or signs of atheromatous involvement of the lower limb diagnosticated by doppler ultrasound.

### Procedures

#### Laboratory measurements

Blood samples were collected as part of our regular CKD patient follow-up, centrifuged and supernatant was stored at −80°C for processing of creatinine, calcium, PO_4_, 25(OH) vitamin D, 1.25(OH)_2_ vitamin D, intact parathyroid hormone (PTH), albumin, high sensitive C reactive protein (hs CRP), fibrinogen, FGF23 and OPG. All measurements were sequentially done with less than one year intervals after freezing.

Creatinine was measured by enzymatic method (Olympus, Rungis, France) using reagents from Randox (Randox, Mauguio, France). Calcium and PO_4_ were assessed by colorimetric method (Olympus, Rungis, France). 25(OH) vitamin D and 1.25(OH)_2_ vitamin D were measured by radioimmunoassay (Immunodiagnostic Systems, Boldon, UK). Intact PTH and insulin were measured by immunoradiometric assay (N-Tact PTH SP IRMA Kit, DiaSorin, Stillwater, MN, USA; Bi Ins IRMA, Cisbio International, Bagnols sur Cèze, France). Albumin was measured by immunonephelometry (Immage Beckman Coulter, Villepinte, France). hs CRP was determined by immunoturbidimetry (Olympus, Rungis, France). Fibrinogen was measured by Von Clauss method (STA Fibrinogen, Diagnostica Stago, Asnières, France). FGF23 was measured with an enzyme linked immunosorbent assay (ELISA) detecting both intact FGF23 and C-terminal fragments (Immutopics International Inc, San Clemente; CA, USA). OPG was determined by ELISA (Biovendor Laboratory Medicine, Brno, Czech Republic) [15], [16].

GFR was estimated using the reexpressed 4-variable Modification of Diet in Renal Disease (MDRD) study equation [17].

Urinary PO_4_ excretion was expressed as Fractional excretion of PO_4_ (FEPO_4_) calculated as follows: FEPO_4_ (%) = urine[PO_4_]×plasma[Creat]×100/urine[Creat]×plasma[PO_4_].

#### Coronary artery calcification imaging

All patients underwent multidetector computed tomography (MDCT).

Regarding data acquisition, all MDCT scans derived from a multidetector-row spiral CT (Lightspeed VCT, General Electric Medical System, Milwaukee, WI, USA). Prospective ECG-triggered step-scan was performed using with 2.5-mm collimation width ×64 detectors so that the center of the temporal window corresponded to 70% of the R-R interval. The scanning parameters were a gantry rotation speed of 0.35 s/rotation, 120 kV and 300 mA 8×2.5 collimation, and 20 mm table feed per rotation. The matrix size was 512×512 pixels and the display field of view was 25 cm. The reconstruction kernel was standard. The temporal resolution was 250 ms.

Regarding image evaluation, calcium score was calculated using a semi-automatically software (Smartscore version 3.5, Advantage Window 4.4 workstation, General Electric, Milwaukee, WI, USA). Coronary calcification was defined as a plaque of > = 4 pixels (area = 1.37 mm^2^) with a density of greater than or equal to 130 Hounsfield units. Quantitative calcium scores were calculated according to the method described by Agatston et al. [18]. Coronary calcium scoring was performed by either a physician or computed tomography technician with specific training for the methodology described above.

### Statistical Methods

Characteristics of the population were described by using proportions for categorical variables and median and range for quantitative variables since their distributions were tested with the Shapiro-Wilk statistic and were mostly skewed.

Associations between the extent of CAC divided into three groups (<100, [100–400[, ≥400) and patient characteristics, vascular risk biomarkers, biological parameters were quantified with odds-ratio (OR) and their 95% confidence intervals (CI). Clinical and sociodemographic variables associated with CAC (at *p*<0.15) were included in multinomial logistic regression models to estimate adjusted OR for biological parameters (especially OPG and FGF23). The cut-off used for CAC has been chosen by respect to CAC scoring: those with CAC<100 (considered as patients with insignificant or mild CAC), those with CAC in the range [100–400[ (presence of moderate CAC) and those with CAC≥400 (presence of severe CAC) [19], [20]. Biological parameters were divided into tertiles. In the case of FGF23 and OPG, patients were divided into two categories, those with a low level (<173.30 RU/mL for FGF23; <10.71 pmol/L for OPG, corresponding to the 1st and 2nd tertiles) and those with a high level (≥173.30 RU/mL for FGF23; ≥10.71 pmol/L for OPG, corresponding to the 3rd tertile).

Spearman correlation coefficient (rho) was used to determine the relationships between quantitative variables.

Significance was set at *p*<0.05. All analyses were carried out with SAS software, version 9.2 (SAS Institute, Cary, NC, USA).

## Results

Clinical characteristics for the 195 CKD patients are summarized in **Table 1**.

**Table 1:** Characteristics of the chronic kidney disease patients.

Parameter	Total
No. of patients	195
Gender, male	112 (57.4%)
Age *years*	70.8 [27.4–94.6]
BMI *kg/m^2^*	26.7 [14.3–47.7]
Smoking habits	95 (49.0%)
Diabetes mellitus	63 (32.3%)
Hypertension	180 (92.3%)
Coronary heart disease	39 (20.0%)
Cerebrovascular disease	14 (7.2%)
Peripheral vascular disease	34 (17.4%)
Vitamin D supplements	
Alfacalcidol	99 (50.8%)
Cholecalciferol	21 (10.8%)
Calcifediol	4 (2.1%)
Statins	64 (37.2%)
Sevelamer hydrochloride	12 (7%)
Erythropoiesis stimulating agents	37 (18.97%)
eGFR (MDRD study equation) (mL/min/1.73 m^2^)	33.2 [6.5–91.9]
>60 mL/min/1.73 m^2^	19 (9.7%)
60-30 mL/min/1.73 m^2^	91 (46.7%)
<30 mL/min/1.73 m^2^	85 (43.6%)
Triglycerides (mmol/L)	1.65 [0.36–4.93]
HDL cholesterol (mmol/L)	1.49 [0.64–3.37]
LDL cholesterol (mmol/L)	2.90 [1.12–6.46]
hs C reactive protein (mg/L)	2.2 [0.1–56.1]
Calcium (mmol/L)	2.38 [1.68–2.73]
Phosphate (mmol/L)	1.07 [0.58–2.34]
Parathyroid hormone (pg/mL)	47.0 [4.0–493.0]
1.25(OH)_2_ vitamin D (pg/mL)	30 [5–80]
25(OH) vitamin D (ng/mL)	19.5 [6.6–76.4]
Hemoglobin (g/dL)	13 [8.9–16.9]
FGF23 (RU/mL)	126.4 [42.9–3415.0]
OPG (pmol/L)	9.1 [2.7–37.6]
CAC	188 [0–3942]

Values were described by using proportions for categorical variables and median [range] for quantitative variables.

Sex ratio of the patients was 112/83 (male/female), median age was 70.8 with a range of 27.4–94.6 years and median BMI was 26.7 [14.3–47.7] kg/m^2^.

Ninety five (49.0%) patients had ever smoked. Diabetes and hypertension were found in 63 (32.3%) and 180 (92.3%) patients respectively. History of CHD was present in 39 patients (20.0%), 14 (7.2%) patients had history of CVD and 34 (17.4%) had history of PVD.

One hundred and twenty four (63.6%) patients were receiving vitamin D supplements, 64 (37.2%) patients were receiving statins, 12 (7.0%) patients were receiving sevelamer HCl, none of them were receiving cinacalcet. Erythropoiesis stimulating agents were administered to 37 (18.97%) patients.

Median eGFR using MDRD was 33.2 [6.5–91.9] mL/min/1.73 m^2^. Median hs CRP was 2.2 [0.1;56.1] mg/L, median intact PTH was 47.0 [4.0–493.0] pg/mL, median 1.25(OH)_2_ vitamin D was 30 [5–80] pg/mL, median 25(OH) vitamin D was 19.5 [6.6–76.4] ng/mL, median FGF23 was 126.4 [42.9–3415.0] RU/mL and median OPG level was 9.1 [2.7–37.6] pmol/L.

Median CAC was 188 [0–3942].

**Figure 1:**
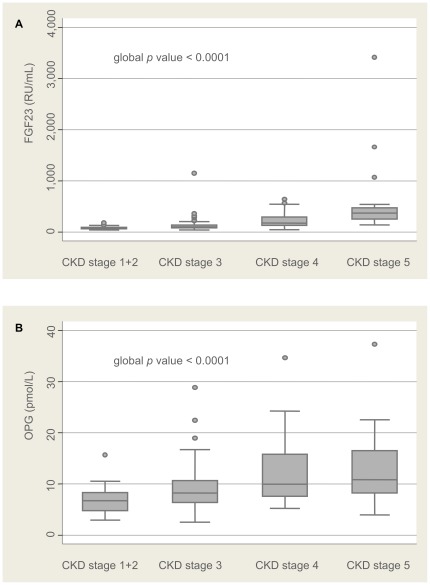
Stratification of bone biomarkers according to CKD stages. FGF23 (A) and OPG (B) median values (quartiles) are depicted according to CKD stages (with a glomerular filtration rate estimated using the reexpressed 4-variable Modification of Diet in Renal Disease (MDRD) 175 study equation).

Stratification of FGF23 and OPG median values (quartiles) according to CKD stages are presented in **Figure 1**.

### Characteristics of the patients according to the level of CAC

Among the 195 patients studied, 79 (40.5%) had insignificant or mild CAC (score below 100), 50 (25.6%) patients had moderate CAC (score in the range [100–400[) and 66 (33.9%) presented severe CAC (score greater than or equal to 400).

The univariate analysis revealed that presence of moderate CAC (score in the range [100–400[) was significantly associated with male gender (OR = 3.17 [1.51;6.65]); age (OR = 2.75 [1.83;4.13] for 10 years increased) and diabetes (OR = 4.48 [1.91;10.5]). Presence of severe CAC (score greater or equal to 400) was significantly associated with the same parameters (male gender: OR = 4.71 [2.30;9.62]; age: OR = 3.04 [2.04;4.51] for 10 years increased; diabetes: OR = 6.08 [2.64;14.0]) including smoking (OR = 3.11 [1.57;6.16]). No significant relationship between therapeutic interventions and moderate or severe CAC was evidenced (**Table 2**).

**Table 2:** Clinical markers associated to increased coronary artery calcifications.

	*Coronary artery calcifications*							*Coronary artery calcifications*	
	*<100 (n = 79)*		*[100–400[ (n = 50)*		*≥400 (n = 66)*			*[100–400[ vs <100*	*≥400 vs <100*
*Variable*	*n*	*%*	*n*	*%*	*n*	*%*	*P-value* *	*OR [CI 95%]*	*OR [CI 95%]*
*Gender*									
*Female*	49	62.03	17	34.00	17	25.76	<.0001	1	1
*Male*	30	37.97	33	66.00	49	74.24		**3.17 [1.51;6.65]**	**4.71 [2.30;9.62]**
*Median Age [min-max]*									
*OR For 10 Years increased*	61.2 [27.4–85.1]		73.4 [45.7–94.6]		75.9 [49.7–86.7]		<.0001	**2.75 [1.83;4.13]**	**3.04 [2.04;4.51]**
*Body mass index*									
*<25*	39	49.37	16	32.00	22	33.33	0.20	1	1
*[25–30[*	28	35.44	21	42.00	30	45.45		1.83 [0.81;4.12]	1.90 [0.91;3.96]
*≥30*	12	15.19	13	26.00	14	21.21		2.64 [0.99;7.01]	2.07 [0.81;5.25]
*Smoking*									
*No*	51	64.56	24	48.00	24	36.92	0.004	1	1
*Yes*	28	35.44	26	52.00	41	63.08		1.97 [0.96;4.06]	**3.11 [1.57;6.16]**
*Diabetes mellitus*									
*No*	68	86.08	29	58.00	35	53.03	<0.0001	1	1
*Yes*	11	13.92	21	42.00	31	46.97		**4.48 [1.91;10.5]**	**5.48 [2.46;12.2]**
*Hypertension*									
*No*	8	10.13	5	10.00	2	3.03	0.26	1	1
*Yes*	71	89.87	45	90.00	64	96.97		1.01 [0.31;3.29]	3.60 [0.74;17.6]
*Dyslipidemia*									
*No*	13	16.67	10	20.00	7	10.77	0.38	1	1
*Yes (LDL ≥ 2.62 mmol/L or statin use)*	65	83.33	40	80.00	58	89.23		0.80 [0.32;2.00]	1.66 [0.62;4.43]
*Vitamin D supplements*									
*No*	27	34.18	18	36.00	25	37.88	0.90	1	1
*Yes*	52	65.82	32	64.00	41	62.12		0.92 [0.44;1.94]	0.85 [0.43;1.68]
*Sevelamer HCl*									
*No*	76	96.20	47	94.00	60	90.91	0.44	1	1
*Yes*	3	3.80	3	6.00	6	9.09		1.62 [0.31;8.35]	2.53 [0.61;10.6]
*Erythropoiesis stimulating agents*									
*No*	66	83.54	43	86.00	49	74.24	0.22	1	1
*Yes*	13	16.46	7	14.00	17	25.76		0.83 [0.31;2.24]	1.76 [0.78;3.96]
*Calcium-based phosphate binders*									
*No*	63	79.75	41	82.00	58	87.88	0.42	1	1
*Yes*	16	20.25	9	18.00	8	12.12		0.86 [0.35;2.14]	0.54 [0.22;1.36]
*Non-calcium-based phosphate binders*									
*No*	77	97.47	47	94.00	61	92.42	0.40	1	1
*Yes*	2	2.53	3	6.00	5	7.58		2.46 [0.40;15.3]	3.16 [0.59;16.8]

* p-value (for variables with more than two categories, the p-value of the test for trend is given).

### Relationships between biological markers of vascular risk and the extent of CAC

Among the biological markers of vascular risk studied, the logistic regression analysis clearly showed high OPG (≥10.71 pmol/L) as the only one variable to be significantly associated with moderate CAC ([100–400[) (OR = 2.73 [1.03;7.26]; p = 0.04) after adjustment for age, gender, diabetes and smoking. Such association failed to persist for CAC scoring higher than 400 (OR = 2.41 [0.91;6.39]). Indeed, severe CAC score was only associated with high FEPO_4_ (≥38.71%) (OR = 5.47 [1.76;17.0]; p = 0.003) and high FGF23 (≥173.30 RU/mL) (OR = 5.40 [1.91;15.3]; p = 0.002) (**Table 3**). Besides, a strong positive correlation was observed between FEPO_4_ and FGF23 whatever the CAC score was (all CAC: rho = 0.53, p<0.0001; CAC<100: rho = 0.45, p<0.0001; CAC [100–400[: rho = 0.51, p = 0.0003; CAC≥400: rho = 0.61, p<0.0001).

**Table 3:** Biological markers associated to increased coronary artery calcifications after adjustment for age, gender, diabetes mellitus and smoking (the p-value of the test for trend is given).

	*Coronary artery calcifications*							*Coronary artery calcifications*	
	*<100 (n = 79)*		*[100–400[ (n = 50)*		*≥400 (n = 66)*			*[100–400[ vs <100*	*≥400 vs <100*
*Variable*	*n*	*%*	*n*	*%*	*n*	*%*	*Global P-value*	*OR [CI 95%]*	*OR [CI 95%]*
*Hemoglobin (g/dL)*									
*<12.5*	23	29.11	16	32.65	22	33.85	0.49	1	1
*[12.5–13.8[*	27	34.18	18	36.73	20	30.77		0.53 [0.17;1.61]	0.37 [0.12;1.16]
*≥13.8*	29	36.71	15	30.61	23	35.38		0.50 [0.16;1.61]	0.52 [0.17;1.62]
*Serum creatinine (µmol/L)*									
*<137*	32	40.51	18	36.00	14	21.21	0.25	1	1
*[137–190[*	26	32.91	14	28.00	24	36.36		0.77 [0.27;2.22]	1.59 [0.55;4.55]
*≥190*	21	26.58	18	36.00	28	42.42		1.76 [0.61;5.13]	**3.17 [1.07;9.37]**
*Calcium (mmol/L)*									
*<2.32*	25	31.65	22	44.00	20	30.30	0.34	1	1
*[2.32–2.44[*	32	40.51	11	22.00	19	28.79		0.53 [0.18;1.53]	1.04 [0.36;2.97]
*≥2.44*	22	27.85	17	34.00	27	40.91		1.16 [0.41;3.32]	2.08 [0.72;6.04]
*Phosphate (mmol/L)*									
*<0.97*	23	29.11	21	42.00	19	28.79	0.46	1	1
*[0.97–1.16[*	28	35.44	18	36.00	23	34.85		0.95 [0.35;2.58]	1.30 [0.47;3.64]
*≥1.16*	28	35.44	11	22.00	24	36.36		0.54 [0.17;1.74]	1.44 [0.46;4.45]
*FEPO_4_ (%)*									
*<25.86*	33	43.42	14	31.11	13	22.03	0.01	1	1
*[25.86–38.71[*	24	31.58	19	42.22	17	28.81		**2.95 [1.01;8.64]**	2.66 [0.86;8.21]
*≥38.71*	19	25.00	12	26.67	29	49.15		2.15 [0.67;6.95]	**5.47 [1.76;17.0]**
*1.25 (OH)_2_ vitamin D (pg/mL)*									
*<23*	23	29.11	18	36.00	18	27.27	0.20	1	1
*[23–38[*	27	34.18	15	30.00	23	34.85		**0.23 [0.07;0.79]**	0.30 [0.09;1.03]
*≥38*	29	36.71	17	34.00	25	37.88		0.33 [0.10;1.05]	0.45 [0.14;1.44]
*25(OH) vitamin D (ng/mL)*									
*<15.7*	24	32.00	19	38.00	20	30.77	0.94	1	1
*[15.7–25.0[*	23	30.67	15	30.00	24	36.92		1.13 [0.39;3.26]	1.53 [0.54;4.40]
*≥25.0*	28	37.33	16	32.00	21	32.31		1.01 [0.36;2.89]	1.22 [0.42;3.49]
*Intact PTH (pg/mL)*									
*<36*	32	40.51	15	30.00	16	24.24	0.35	1	1
*[36–75[*	26	32.91	20	40.00	20	30.30		1.40 [0.50;3.94]	1.11 [0.38;3.24]
*≥75*	21	26.58	15	30.00	30	45.45		1.15 [0.39;3.36]	2.10 [0.74;5.97]
*FGF 23 (RU/mL)*									
*<173.30*	58	73.42	36	72.00	35	53.03	0.003	1	1
*≥173.30*	21	26.58	14	28.00	31	46.97		2.05 [0.71;5.92]	**5.40 [1.91;15.3]**
*Osteoprotegerin (pmol/L)*									
*<10.71*	65	82.28	27	54.00	37	56.06	0.11	1	1
*≥10.71*	14	17.72	23	46.00	29	43.94		**2.73 [1.03;7.26]**	2.41 [0.91;6.39]

### Prevalence of high OPG and FGF-23 levels according to the extent of CAC

**Figure 2:**
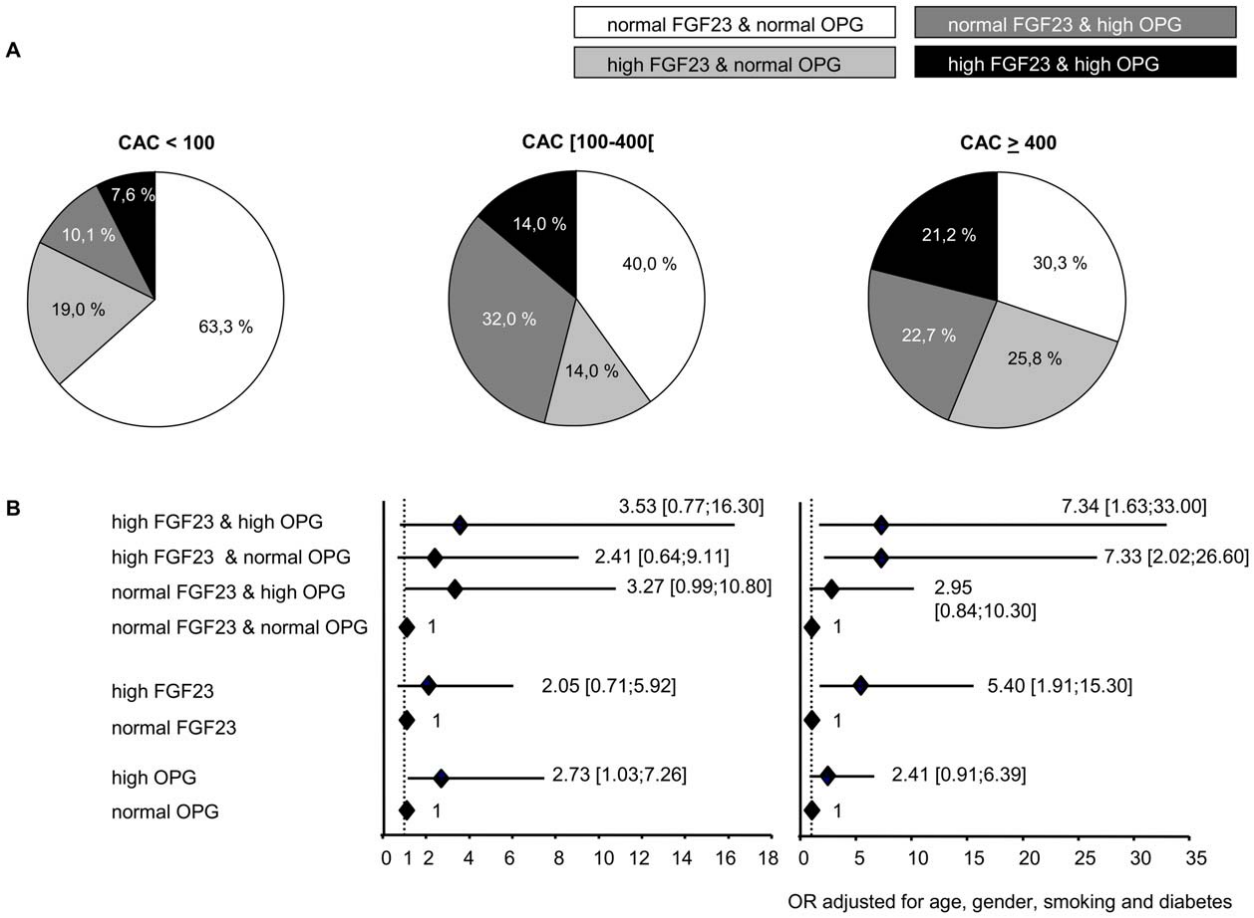
Fibroblast growth factor 23 and Osteoprotegerin are differently associated to the severity of CAC. (A) Proportions of patients with normal (<173.30 RU/mL) or high (≥173.30 RU/mL) fibroblast growth factor 23 (FGF23) and normal (<10.71 pmol/L) or high (≥10.71 pmol/L) osteoprotegerin (OPG) levels within each coronary artery calcification (CAC) category. Sections of pie charts indicate proportions of patients with FGF23 and/or OPG levels by CAC category. (B) Prevalence rate ratio of CAC associated with increased levels of FGF23 and/or OPG levels.

To further compare the relative prevalence of abnormally high (greater than the lower bound of third tertile) OPG versus FGF23 levels across the spectrum of CAC, the proportions of participants with normal or high OPG and FGF23 values within each category of CAC were determined. (**Figure 2A**). As expected, the predominant pattern in the range of insignificant or mild CAC (<100) was normal FGF23/normal OPG (63.3%). In the range of moderate CAC, an isolated increase in OPG (46.0%) was the most common pattern and was more likely than an isolated increase in FGF23 (28.0%). By contrast, at the level of severe CAC, the most common pattern was characterized by an isolated increase in FGF23 (47.0%).

### Relationships between OPG/FGF23 (as a composite criterium) and the extent of CAC

Logistic regression model clearly showed that, in these patients, the risk to present severe CAC when FGF23 level was high (≥173.30 RU/mL) was not significantly different when OPG was normal (OR = 7.33 [2.02;26.60]) or high (OR = 7.34 [1.63;33.00]). Conversely, even though only a tendancy exists, the risk to present moderate CAC when OPG level was high (≥10.71 pmol/L) was not significantly different when FGF23 was normal (OR = 3.27 [0.99;10.80]) or high (OR = 3.53 [0.77;16.30]) (**Figure 2B**).

## Discussion

In this study, clinical variables such as age, diabetes mellitus and smoking habits were associated with CAC in our population of ND-CKD patients. Among biomarkers of vascular calcifications, our results clearly confirmed that both high OPG and FGF23 levels were associated with CAC in this population. Original findings of our study are that association of both markers with CAC depends on the severity of CAC extension: OPG is associated to moderate CAC while FGF23 rather represents a biomarker of severe CAC in these patients.

Previous studies have reported an association between high OPG levels and CAC in CKD (in particular one from our group) [6], HD [7], [10], [21] and diabetic patients [22], [23]. Here, we demonstrated for the first time that high OPG is only associated to moderate rather than severe coronary calcifications. These interesting results are in total agreement with a previous study from Morony et al. [24] in a model of atherogenic diet-fed *ldlr* ^(−/−)^ mice. Indeed, the authors clearly showed that plasma OPG level increased with initiation of the atherogenic diet, reached a maximum value after one month of diet and did not increase further during the four following months while aortic atherosclerosis lesions were still progressing. In our study, the same pattern is observed. OPG values are significantly increased in patients with moderate ([100–400[) compared with mild (<100) CAC, this increased level being comparable with that of patients presenting severe (>400) CAC. This clearly suggests that OPG appears as a marker of atherosclerosis/vascular calcification onset rather than its severity or progression. Considerable controversy exists regarding the role of OPG in the development and progression of vascular calcifications. However, the only theory we can speculate on is that OPG is increased in response to vascular insult as the component of a complex compensatory mechanism, probably secondary to inflammatory processes. Indeed, proinflammatory mediators, particularly TNF-alpha, which participate to the development of vascular calcification through induction of alkaline phosphatase can also stimulate OPG synthesis in vascular smooth muscle cells and endothelial cells [25]–[27] in an attempt to possibly counteract osteogenic or pro-apoptotic calcification mechanisms. However, further in vitro and in vivo studies need to be conducted in order to elucidate the exact implication of such molecule in the early progression of vascular calcifications.

Regarding FGF23, although its association with CAC was evidenced in HD [8], [9], this relationship was not clear in all studies related to ND-CKD patients. In their analysis, Gutierrez et al. [11] could describe an association between FGF23 and CAC. However, this association was no longer significant after multivariable adjustment or when examined on a continuous scale. The recent study from Desjardins et al. [28] clearly demonstrated in 142 ND-CKD patients an association between high FGF23 and aortic calcifications and to a lesser extent an association with CAC in a subgroup of 93 patients. As previously observed by Gutierrez et al., FGF23 was also no longer associated to CAC in their multivariate analysis. The authors speculated that, contrary to aortic calcifications, the lack of association between CAC and FGF23 may be explained by the different type of calcifications observed (intimal for coronaries and medial in the case of aorta): FGF23 being more related to mineral metabolism disturbances, would favor medial rather than intimal atheromatous calcifications. Actually, the persisting association between CAC and FGF23 after full adjustment, observed here, in more than twice the number of patients, may probably reflect a power limitation of their study. Desjardins et al. also depicted correlations between aortic as well as coronary calcifications and FGF23. In the present study, we were not able to evidence any correlation, as our results clearly demonstrated an association between FGF23 and severe rather than moderate CAC. Interestingly, these latter findings were also described in the study from Jean et al. in HD patients [10].

The potential evidence of high FGF23 as a biomarker of severe CAC in our study cannot lead to the conclusion of a real role of FGF23 in the pathogenesis of CAC. Indeed, it was initially postulated that FGF23, due to its phosphaturic and hypophosphatemic actions [29], may be considered as a protective molecule against vascular calcifications. During decline in renal function, the commonly observed rise in FGF23 should probably reflect an increased production by osteocytes to help maintening normophosphatemia rather than a lack of removal by the kidney [30]. However, a previous work in this population could report a preponderance of C-terminal fragments (secondary to proteolytic cleavage of intact FGF23) suggesting that less than one-quarter of the circulating FGF23 was bioactive in these patients [31]. In addition, the original work from Goetz et al. [32] could provide evidence that proteolytic cleavage of this molecule abrogated its activity by removing FGF23's binding site for the FGF receptor-Klotho complex and also by generating an endogenous inhibitor of FGF23. Therefore, taken into account all these findings, it could be postulated that the high levels of FGF-23 (both intact and C-terminal fragments) measured in our population might reflect a lack of efficiency rather than an excess activity of this phosphatonin. However, importantly we observed a significant association between high levels of FEPO_4_, the first target of FGF23, and CAC. Finally, as no association was reported between phosphatemia and CAC in our study, we may also assume that such association between FGF23 and CAC involves phosphate-independent mechanismes. Indeed, the recent report of Faul et al. [33] in an animal model strongly suggests that FGF-23 *per se* is able to induce an impairment in cardiac function. Besides, the lack of association observed here between serum phosphate and CAC is surprising but not unexpected as previous studies also reported similar findings in predialysis patients [2], [34]. Russo et al. suggested that a single measurement of serum phosphate could not reflect the lifetime exposure due to rapidly changes over time for compensatory mechanisms that are mostly active early in the course of CKD [35].

Our study acknowledged some limitations. Both intact FGF23 and C-terminal fragments have been measured here. Even though several studies reported strong correlations (r = 0.97; p<0.0001) existing with the active intact FGF23 [36], this assay does not exactly reflect the biologically active form of FGF23. However, it is to note that a strong positive correlation (rho = 0.52731; p<0.0001) was observed here between C-terminal fragments and FEPO_4_, the intact FGF23 first target. Circulating klotho levels are missing due to the lack of availability of assays in plasma or urine. The relatively small sample size of this study may have prevented some of the detected associations from being statistically significant. Finally, the cross-sectional nature of this study can not clearly evidence the kinetic of OPG and FGF23 over the progression of coronary calcifications, a prospective study being strongly required.

In conclusion, even though both OPG and FGF23 constitute strong predictors of mortality in HD patients, their relationships to vascular calcifications are different, especially in ND-CKD population. Our results clearly showed that both high OPG and FGF23 levels were associated with CAC in this population of ND-CKD patients, OPG being nevertheless a stronger associated factor of moderate CAC while FGF23 rather represents a biomarker of severe CAC.
